# Barriers and enablers in the micronutrient powder supply chain: lessons from a process evaluation of a home fortification programme in Bangladesh

**DOI:** 10.3389/fnut.2024.1429526

**Published:** 2025-01-06

**Authors:** Md. Fakhar Uddin, Md. Aminul Islam, Mahfuzur Rahman, Tahmeed Ahmed, Haribondhu Sarma

**Affiliations:** ^1^Nutrition Research Division, International Centre for Diarrheal Disease Research, Bangladesh (icddr,b), Dhaka, Bangladesh; ^2^School of Health and Rehabilitation Sciences, The University of Queensland, St Lucia, QLD, Australia; ^3^National Centre for Epidemiology and Population Health, The Australian National University, Canberra, ACT, Australia

**Keywords:** barriers, enablers, micronutrient powder, supply chain, Bangladesh

## Abstract

**Background:**

Bangladesh Rural Advancement Committee (BRAC), a leading non-governmental organization (NGO), implemented a large-scale Home Fortification (HF) with Micronutrient Powder (MNP) programme from 2013 to 2018 aimed to reduce undernutrition and iron deficiency anemia among children aged below 5 years old. An adequate and timely supply of MNP was crucial for successful implementation of the programme, but very few studies have documented implementers’ MNP supply chain experiences. Therefore, this study aimed to explore the barriers and enablers in the MNP supply chain in Bangladesh.

**Methods:**

We conducted this process evaluation in five rural sub-districts and three urban slums from March 2016 to February 2017. We conducted 15 Key Informant Interviews (KIIs) with HF programme personnel, 41 In-depth Interviews (IDIs) with direct HF programme implementers and reviewed relevant documents. We analyzed data using thematic and root-cause analysis approaches.

**Results:**

Participants reported the barriers in the MNP supply chain included lack of raw materials for MNP production by local manufacturer, political unrest and insufficient transport facilities, a lack of space for MNP buffer stock at BRAC’s central warehouse, and coordination gaps between BRAC’s national and sub-national level staff. Enablers to each of the barriers mentioned include ensuring buffer stock at all levels, raising separate transport requisition for MNP supply, and recruiting dedicated supply chain officers.

**Conclusion:**

Concurrent course-corrections based on process evaluation findings improved MNP supply chain performance, resulting in higher MNP sales and coverage. The identified barriers and enablers provide useful insights for similar programs, emphasizing the importance of a resilient and well-managed MNP supply chain.

## Background

In low and middle-income countries (LMICs), infants and young children’s nutritional needs are difficult to meet due to high costs, limited availability, and lack of access to nutrient-rich foods (1). To address this, the World Health Organization (WHO) recommends the use of low-cost micronutrient powder (MNP), a combination of vitamins and minerals provided in a single-dose sachet that needs to be added into a child’s meal once daily, just before consumption (2). WHO suggests MNP distribution in regions with high childhood anemia rates (>20%) to improve children’s nutritional status (3). Therefore, home fortification with MNP programs have been implemented internationally (4, 5). Despite these initiatives, only one-fifth of the targeted 15 million children worldwide received MNP in 2014 (6). Meeting the MNP distribution targets on time may be challenging due to barriers in the MNP supply chain.

A supply chain is an integrating process involving manufacturers, suppliers, distributors, and retailers, working collaboratively to acquire raw materials, produce final products, and then distribute these products to retailers and customers (7, 8). Supply chain constraints are acknowledged as factors affecting programs outcomes and timely solutions to the constraints is crucial for achieving significant improvements in program effectiveness, scale, and impact (7, 9, 10). While MNP in LMICs is predominantly sourced from international suppliers; however, with substantial investments, local production may be feasible to ensure uninterrupted and continuous supplies (10, 11), increase MNP sales and coverage (12, 13). However, the barriers and enablers in the MNP supply chain have yet to be explored in-depth, particularly in the context of Bangladesh.

Consistent disruptions in the MNP supply chain, caused by funding delays, supplier capacity constraints, and lengthy procurement process for manufacturing and shipping, have been documented in various settings (11, 13). In Vietnam, the MNP supply chain was disrupted due to a lack of transportation, underestimation during forecasting, and payment delays, leading to unavailability of MNP at local health centers and among health workers (8, 14). The Bangladesh Rural Advancement Committee (BRAC), a leading non-governmental organization (NGO) in Bangladesh, implemented a large-scale HF with MNP programme from July 2013 to December 2018 by providing MNP known as *Pushtikona-5* at the community or household level through its frontline volunteer health workers to reduce undernutrition and iron deficiency anemia among children below 5 years old. BRAC received technical assistance from the Global Alliance for Improved Nutrition (GAIN) to implement the HF with MNP program. The MNP was manufactured by a local company called Renata Limited. However, our previous qualitative assessments revealed MNP shortage at BRAC’s local offices and among frontline health workers due to persistent supply chain challenges during the implementation of a large-scale Home Fortification with MNP program (14, 15).

Very few studies have explored implementers’ MNP supply chain experiences during HF programme implementation. To address this knowledge gap, BRAC and the Children’s Investment Fund Foundation (CIFF) contracted the International Centre for Diarrheal Disease Research, Bangladesh (icddr,b) to conduct a process evaluation (PE) as part of a concurrent evaluation of the HF with MNP programme (16). Therefore, this process evaluation aimed to explore the barriers and enablers in the MNP supply chain in Bangladesh. By thoroughly documenting the MNP supply chain experiences, this process evaluation findings facilitated concurrent course corrections, enhancing supply chain performance and contributing valuable lessons for designing effective and efficient MNP supply chain systems in similar contexts.

## Materials and methods

### Study design

We conducted this process evaluation from March 2016 to February 2017 and collected data both retrospectively and prospectively to capture the full range of MNP supply chain implementation processes. Qualitative data were collected through fact-checking interviews, in-depth interviews (IDIs), key informant interviews (KIIs), and document reviews. Fact-checking interviews were useful for verifying and validating information, whereas IDIs and KIIs provided deeper insights into the perspectives and experiences of those involved in the MNP supply chain.

#### Conceptualization of the process evaluation

**FIGURE 1:**
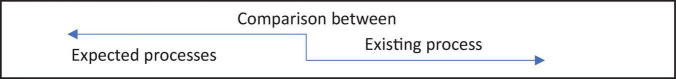
Conceptualization of the process evaluation. Illustrates the conceptualization of the process evaluation to examine the comparison and identify gaps between the “expected process” (planned activities) and the “existing process” (current implementation practices) in the micronutrient supply chain to guide improvement through this process evaluation’s findings.

We conceptualized this process evaluation as a comparison between the expected and existing MNP supply chain processes to identify gaps that were influencing the current HF programme implementation (Figure 1). In this figure, “Expected process” denotes planned activities, whereas “Existing process” denotes the programmer’s actual or current implementation practices.

**FIGURE 2:**
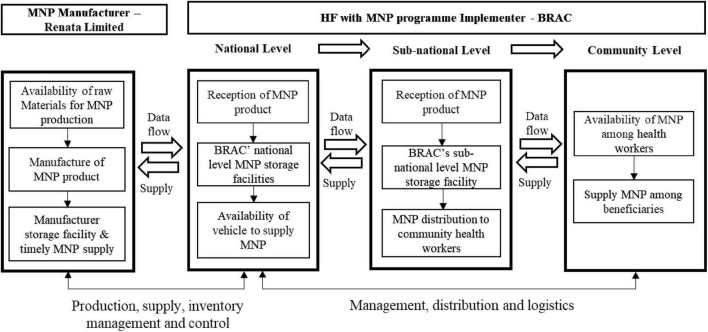
Conceptual framework for investigation of the micronutrient power supply chain. The framework highlights key micronutrient powder supply chain milestones, detailing Renata Limited’s role in production and timely supply, and BRAC’s responsibilities in receipt, storage, and distribution across at the national, sub-national, and community levels.

In the process evaluation of BRAC’s Home Fortification (HF) with MNP program, a conceptual framework (Figure 2) was developed to systematically investigate the management of the MNP supply chain. This framework was developed after in-person group discussions with program implementers (including BRAC HF Programme Managers, Sector Specialists and Product Managers of Renata Limited). This framework outlined the key milestones of MNP supply chain necessary to achieve the program’s goals related to MNP sales, coverage, and usage targets. It identified specific roles and responsibilities for both the manufacturer (Renata Limited) and BRAC at each stage of the supply chain. For example, the manufacturer planned to ensure the availability of MNP raw materials and the timely production of MNP in order to supply MNP to BRAC on time. BRAC was also expected to meet other milestones, such as timely receipt, storage, and distribution of MNP at the national, sub-district, and community levels. The process evaluation examined various barriers and enablers related to achieving these MNP supply chain milestones, particularly focusing on how storage and distribution conditions affected the overall effectiveness of the MNP program.

#### Investigation steps of the process evaluation

This process evaluation was carried out by following some interrelated investigation steps for a comprehensive understanding of MNP supply chain management within the HF program. First, we developed a conceptual framework (Figure 2) that outlined broad areas of investigation in the process evaluation. This served as a guide for understanding key milestones in the MNP supply chain. Then, we conducted fact-checking interviews with 16 key HF program staff to assess whether the MNP supply chain was functioning as intended. These interviews helped in tracking the progress at each milestone and evaluating how internal and external factors (e.g., raw material availability, logistics, storage) influenced the supply chain process. The initial findings from the process tracking activities were shared with national-level personals from BRAC, GAIN and Renata Limited in formal periodic stakeholder meetings, which included presentations and discussions. This collaborative approach allowed stakeholders to identify and prioritize the most critical issues for further investigation, ensuring that the evaluation focused on the most pressing challenges or barriers affecting MNP supply chain. The collaboration focused on co-developing data collection tools, aligning study objectives with stakeholder priorities, and incorporating feedback iteratively. Following stakeholder input, we conducted an in-depth investigation into the factors affecting the MNP supply chain. Throughout the MNP supply chain, we planned to conduct a series of qualitative interviews with purposively selected key stakeholders, including warehouse managers, frontline volunteer community health workers (CHWs) namely Shasthya Sebika, and product managers at the national and sub-national levels. Finally, we analyzed the collected data using thematic and root-cause analysis methods to uncover the underlying factors contributing to MNP supply chain inefficiencies. This step helped clarify where the supply chain was breaking down and which strategies could be refined or adjusted.

#### Study sites

We selected five rural *Upazilas* (sub-districts) as study sites in Bangladesh, namely *Mithapukur of Rangpur district, Shalikha of Magura district, Saturia of Manikgonj district, Fulgazi* of Feni district and, *Feni Sadar Upazila of Feni district*, along with three urban slums in Dhaka (the capital city of Bangladesh), namely *Manda, Begun bari* and *Karail slums*. The areas were selected based on MNP sales performance and geographical diversity.

#### Recruitment of study participants and data collection

We purposefully selected interviewees involved in selling and distributing MNP to gather a wide range of perspectives and detailed insights into the MNP supply chain. In the community level, we conducted 21 in-depth interviews (IDIs) with BRAC’s Shasthya Sebikas and 11 with Shasthya Kormis (next-level supervisors of Shasthya Sebikas). We conducted IDIs with 9 BRAC’s Programe Officers (next-level supervisors of Shasthya Kormis), and 5 with Officer Supply Chain and Quality Assurance at the sub-national level to explore root causes influencing the MNP supply chain. These participants were involved in the MNP supply chain process from national to sub-national levels and the distribution of MNP to Shasthya Sebikas for sale to programme beneficiaries. To supplement these findings, we conducted Key Informant Interviews (KIIs) with five Upazila Managers, three Branch Managers, and five District Managers in the sub-national level. These participants were knowledgeable and responsible for ensuring MNP availability, raising requisitions, and distributing MNP at the sub-national level on time.

We conducted IDIs at the national level with three Sector Specialists and one Programme Manager for BRAC’s HF programme, and one MNP Product Manager from the local manufacturer, Renata limited. They were directly involved in the production and supply of MNP from national to sub-national levels. To supplement the IDIs data and to gain a comprehensive understanding of MNP supply chain dynamics, we conducted KIIs with one Senior Manager-Human Resources from BRAC and one Program Manager from GAIN. They had extensive knowledge of MNP production, storage, and supply, and played important roles in distributing MNP and recruiting HF supply chain focal points for the MNP program. We also reviewed relevant documents, including BRAC’s HF programme implementation proposal, program registers, MNP calendar, interpersonal communication materials, quarterly progress reports from BRAC and GAIN, sales reports from sub-district offices, previous research reports from icddr,b and a published article (14).

We conducted interviews at the respondents’ convenient time and location by using flexible semi-structured guidelines and audio recorders. On average, each interview lasted 65 min. The number of interviews was determined by achieving data saturation. A well-trained team with extensive prior experience in qualitative data collection, led by study’s Principal Investigator (PI), ensured data consistency and quality.

#### Data analysis

We transcribed verbatim the audio-recorded interviews and coded textual data using ATLAS.ti (version 5.2) software. The data analysis steps involved refining and organizing codes to accurately capture themes and patterns emerging from the data. Two independent data coders were involved and resolved any discrepancies in the coding process, and ensured accuracy and consistency. The software-generated coded outputs were organized and displayed into a matrix table to facilitate data reduction. Thematic analysis was conducted to identify patterns and themes relevant to the MNP supply chain. Additionally, we applied a root-cause analysis approach to delve deeper into the identified barriers and enablers, exploring the “why” behind these influences to pinpoint their root causes. We selected root-cause analysis to complement thematic analysis by providing a structured approach to identifying the underlying causes of the barriers and enablers faced in the MNP supply chain. This dual approach provided a comprehensive understanding of both immediate and underlying issues, making the findings actionable for stakeholders aiming to improve the MNP supply chain. To enhance the reliability and validity of our findings, we cross-checked the analysis extensively within and between themes and sub-themes. Verbatim quotes were presented to demonstrate diverse participant perspectives, providing a nuanced view of participants’ experiences and insights.

#### Ethical approval

Prior to study implementation, the Research Review Committee and Ethical Review Committee of icddr,b approved the study (Protocol number: PR-13101). Before conducting interviews, we took written informed consent from study participants prior to conducting interviews.

## Results

**FIGURE 3:**
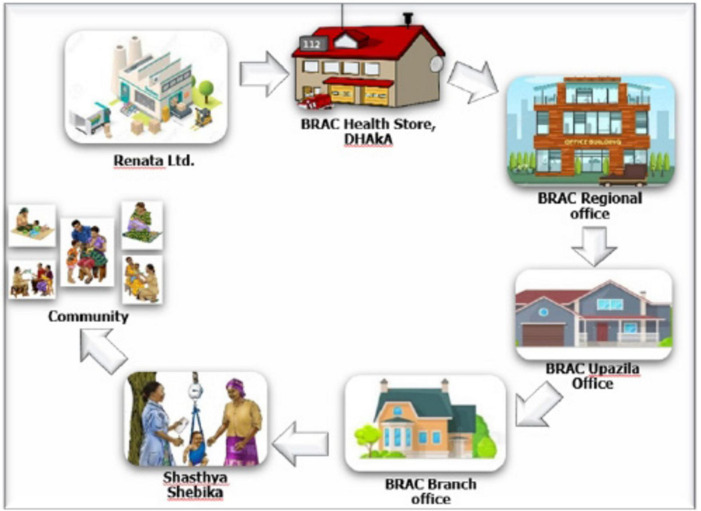
Micronutrient powder supply network. The figure illustrates the MNP supply chain, starting with production by Renata Limited, followed by distribution through BRAC’s central, regional, and sub-district/Upazila offices, then to community/branch office-level stores, and finally delivered by Shasthya Shebikas to beneficiaries.

To visually represent the expected MNP supply chain network, Figure 3 outlines the distribution process from production to beneficiaries, based on HF program stakeholders’ inputs. The supply chain begins with Renata Limited, responsible for producing and packaging MNP, which was then sent to BRAC’s national warehouse as the central hub. From there, MNP was distributed to BRAC’s sub-national offices, including district and sub-district stores, which supply frontline health workers, known as Shasthya Sebikas. These health workers serve as last-mile distributors, delivering MNP to households and communities, ensuring children under five receive the product through community-based sales or distributions.

**FIGURE 4:**
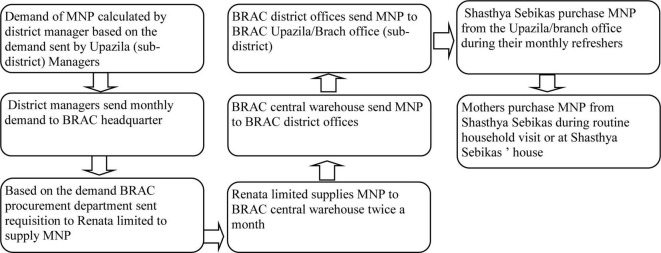
Planned micronutrient powder requisition, distribution and selling process. The figure shows BRAC’s MNP requisition, distribution and sales process, highlighting efforts to improve availability and supply chain efficiency through demand forecasting and monthly meetings with Shasthya Sebikas.

Participants from BRAC’s HF with MNP program reported that the primary objective was to improve MNP product availability and streamline the supply chain efficiency by accurately forecasting sub-district level MNP needs. To achieve this, BRAC facilitated monthly meetings with the MNP manufacturer to confirm and adjust orders based on demand forecasts, with technical support provided by GAIN. Figure 4 illustrates the expected MNP requisition and sales process. Key barriers and enablers within the MNP supply chain, influencing the HF program’s success are detailed in the following paragraphs.

**FIGURE 5:**
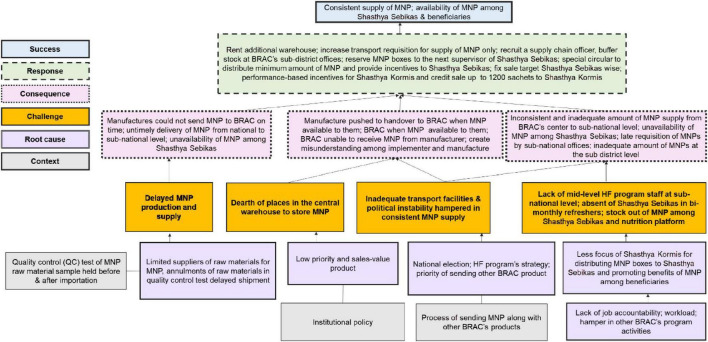
Root-cause analysis of challenges and success in the micronutrient powder supply chain. It uses different color-coded boxes to represent the MNP supply chain challenges, their root causes, context, consequences, responses, and successes. This Figure summarizes challenges such as delays, stockouts, and lack of transport facilities, along with their root causes (e.g., manufacturing delays, political instability), consequences of these challenges, including disruptions in MNP availability, and the responses implemented (e.g., increasing storage capacity, enhancing transport, and hiring more staff) to contribute to the success of ensuring consistent MNP supply.

Figure 5 below depicts the MNP supply chain barriers, enablers and its root-causes. The challenges identified include delays in manufacturing and supply, inadequate storage and transport facilities, political instability, limited staff and accountability, raw material quality issues, and competing program priorities, all of which led to supply disruptions from manufacturers to beneficiaries. In response, interventions such as renting additional storage, recruiting supply chain officers, expanding transport capacity, and introducing performance-based incentives were implemented to ensure consistent MNP availability, improve supply and access for community health workers and beneficiaries. Detailed insights from program implementers and beneficiaries are explained in the following sections.

### Barriers and enablers in the MNP supply chain

#### Lack of raw materials to produce MNP

Participants at the national level stated that the local manufacturer, Renata limited faced challenges to produce the required number of MNP sachets due to shortage of imported raw materials for MNP production. This hindered consistent supply from Renata limited to BRAC. The root-causes of this shortage included delayed shipments from international suppliers, rejection during quality control (QC) testing, and limited storage capacity at the manufacturing company. Participants, for example, stated that when imported raw materials failed quality control tests in Bangladesh, it triggered a lengthy procurement process, time loss and delayed MNP production. For this reason, BRAC was unable to send MNP on time to the sub-national level for distributing among frontline community health workers.

*“Due to delays in receiving raw materials for MNP production from international suppliers, Renata limited was unable to supply MNP at times or supplied insufficient amount of MNP sachets to BRAC. For example, from July to September 2015, BRAC received 8,242,710 sachets of MNP from Renata limited, despite having ordered 12,628,800 sachets. As a result, BRAC either sent none or fewer MNP sachets to field offices, resulting in shortage of MNP among Shathya Shebekas.”* **KII-04, National level participant**

**FIGURE 6:**
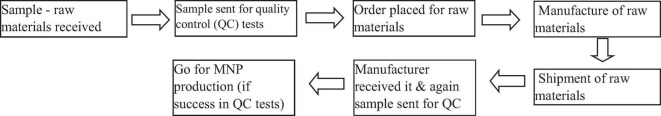
Steps for procurement of MNP raw materials. It outlines the MNP raw material procurement process, begins with Renata Limited sourcing materials from foreign suppliers, followed by submitting samples for initial government quality control testing, shipment and final approvals from government and then proceed for MNP production.

The procurement process for MNP production raw materials, as illustrated in Figure 6 based on participants’ explanations, begins with Renata Limited sourcing materials from foreign suppliers. Upon receiving these raw materials, initial samples were submitted to the government for quality control testing. Once the government provided initial approval, Renata placed orders for the required quantities. After the materials are delivered, they undergo another round of quality testing for final approval. Only after passing these rigorous tests does Renata proceed with MNP production.

The recurrent nature of these supply chain interruptions contributed to the perceptions among stakeholders that such production issues were normal and unresolved, impacting the program’s ability to consistently deliver MNP to children in need. To address this challenge, one BRAC HF with MNP programme personnel said:

*“BRAC implemented strategic MNP procurement measures in 2016 and 2017, when imported raw materials became more readily available to the manufacturer, Renata limited. These measures included procuring larger quantities of MNP sachets from Renata limited and maintaining a buffer stock of MNP sachets at BRAC’s central stores. These measures prevented MNP shortages during periods of high MNP demand and raw materials scarcity that result in positive effects on BRAC’s consistent and timely supply of MNP at the national and sub-national levels.”* KII-06, BRAC HF with MNP personnel

#### Limited storage capacity for MNP sachets in central warehouse

BRAC national level personals reported that initially, ensuring the timely supply of MNP sachets from the national to sub-national levels was deemed not feasible due to stockouts in BRAC’s central warehouse. Three participants reported that BRAC faced difficulties in storing large quantities of supplied MNP sachets from Renata limited due to BRAC’s limited storage capacity. Renata limited, for instance, was reluctant to store surplus sachets and pushed BRAC to receive all MNP sachets, because MNP had a lower profit margin compared to other products, making it less of a priority. According to one participant, BRAC ordered 12,628,800 MNP sachets between October and December 2015, but Renata limited only supplied 13,844,670 sachets. Furthermore, BRAC had limited central warehouse capacity to store MNP, as they had other prioritized programs’ products for storage, so MNP sachets were stored subject to availability of space.

“*In 2014, there was a store in Uttara with a limited capacity for saline, reading glass and MNP. So, we were unable to receive MNP from Renata limited as requested. For example, we requested 40 lacs (4 millions) MNP sachets but only received 10 lacs (1 million) due to our limited storage capacity. Given this situation, BRAC hired a separate store exclusively for MNP products in 2015 to ensure consistent MNP supply at the national and sub-national levels.”* IDI-05, BRAC MNP programme personnel at national level

BRAC applied several strategic steps to resolve the MNP storage problem by renting a separate central-level store with the capacity of storing 4 million MNP sachets and four regional warehouses (with a capacity of storing 2 million MNP sachets each). Moreover, a new program assistant was hired to monitor storage issues. The program assistant coordinated between central store and sub-national offices in terms of ensuring the availability of MNP at the central store and timely distribution to the sub-national offices in accordance with requisitions. These storage strategies enabled BRAC to receive larger supplies of MNP sachets from Renata limited in 2016, which reduced MNP stock outs at the national, sub-national, and community levels.

#### Political unrest and, insufficient transportation facilities

Three participants from BRAC head office reported that BRAC faced challenges in ensuring a timely and consistent supply of MNP sachets from national to sub-national levels due to frequent transport strikes during political unrest, particularly surrounding the 2014 national election. The disruptions severely affected BRAC’s ability to distribute MNP sachets to field offices. Moreover, BRAC had limited transportation capacity and had no separate transport requisition provision specifically for sending MNP, which compounded delays in MNP supply. MNP sachets were often loaded into trucks only if space was available after loading other BRAC products, leading to further delays in MNP supply delays.

*“We were unable to send MNP sachets from BRAC head office to sub-district level offices on time. So, Shasthya Sebikas were in shortage to sell and distribute MNP to beneficiaries. This occurred as a result of transport strike during the political crisis preceding the 2014 national election.”* IDI-02, BRAC MNP programme personnel at national level

In 2016, BRAC and GAIN addressed these transportation challenges by securing additional funding from donors involved in the HF program. This funding enabled the creation of a dedicated transport budget specifically for MNP sachets, resolving the previous delays caused by space-sharing in vehicles with other BRAC products.

*“Initially, it was challenging to supply MNP to the rural areas on time. Because, when other programs asked for cars to transport their products, we requested them to transport MNP alongside other products. The number of vehicles was also limited to transport other products. Donors provided financial support in the second year of the HF program’s implementation to maintain a separate budget for raising separate requisitions for supply MNP. We can now send MNP to our field offices as soon as Renata’s supply arrives.”* IDI-05, BRAC MNP programme personnel at national level

In addition to transportation improvements, BRAC also implemented a buffer stock strategy in 2016 to mitigate the risk of stockouts caused by unanticipated political unrest or transportation delays. Participants reported that BRAC sent 50% more sachets than requested to sub-district offices, ensuring that a reserve supply of MNP was always available. This strategy helped maintain MNP supply continuity and reduced stockouts among Community Health Workers (CHWs).

*“Now, if a sub-district office requests 1 lac MNP sachets, we send 1.5 lac sachets to ensure MNP remains after distribution to Shasthya Sebikas. This buffer stock ensures consistent MNP supply and reduce MNP stockout among Shasthya Sebikas.”* **IDI-05,** BRAC MNP programme personnel at national level

#### Lack of adequate support from focal person at the sub-national level

Eight participants from BRAC’s national and sub-national levels mentioned that BRAC initially faced challenges in supplying MNP sachets to sub-national offices and targeted households on time due to delays in requisitions from local offices to headquarters. These delays were attributed to the heavy workload of sub-national BRAC’s personnel and a lack of motivation and accountability toward the HF with MNP program activities. Two participants stated that BRAC’s sub-district Managers did not feel pressured by their supervisors to submit MNP requisitions on time, because their local supervisors prioritized other BRAC programs activities from where their salary came from, which had a direct impact on the MNP supply chain, causing its shortage at the household’s level. Three other participants reported that some sub-district Managers requested fewer MNP sachets than required. They were concerned that distributing MNP sachets would burden Shasthya Sebikas, increase their workload, and negatively affect the implementation of other BRAC programs. As a result, MNP shortages occurred among Shasthya Sebikas, leading to 72% of caregivers running out of MNP at the household level.

*“The Upazila [sub-district] Manager used to send a request to our head office for a smaller quantity of MNP sachets to avoid the hassles of selling MNP and easily meet their monthly MNP sales target. Upazila Manger once told me that Shasthya Sebikas selling more MNP sachets could disrupt their regular activities, making it difficult to instruct Shasthya Sebikas to carry out other programme activities as planned.” Key Informant Interview at the sub-national level, KII 05*

Two national-level HF programme staff reported that in 2016, BRAC hired ten Supply Chain and Quality Assurance Officers at the sub-national level to address above-mentioned challenges. The officers were responsible for estimating the demand for MNP sachets and submitting timely requisitions to BRAC headquarters to ensure consistent supply at the sub-district level. Moreover, they identified root causes of MNP stockouts through field visits and worked closely with headquarters staff, communicating via email and mobile phone to address supply gaps. They also facilitated the inter-shifting of MNP sachets between sub-district (Upazilas) with excess MNP. This adaptive strategy helped to balance MNP distribution in some regions while addressing surpluses in other sub-districts. Thus, by the third year of implementation “in 2016,” these strategic changes had significantly improved the MNP supply chain, reducing stockouts and ensuring that Shasthya Sebikas had adequate supplies to distribute to beneficiaries on time.

*“We initially struggled to supply MNP as we had no direct coordination mechanism and requisition submission with BRAC headquarters for timely MNP supply. In 2016, 10 supply chain officers were hired for the sub-national level to ensure timely MNP requisitions from the field level and subsequent delivery by the head office to the field.”* In-depth interview at the sub-national level, IDI-03

#### Individual and programmatic influences

Six participants from BRAC sub-district offices reported that during regular bi-monthly refresher training sessions, they found it difficult to provide MNP sachets to Shasthya Sebikas because some Shasthya Sebikas did not attend the training and some other refused to sell MNP in favor of other high-demand and profitable BRAC products. Additionally, limited transportation allowances, particularly for Shasthya Sebikas in remote or hard-to-reach areas, illness, and family obligation further discouraged their attendance.

*“Last month, 612 of 923 Shasthya Sebikas attended our HF refresher training, while the remaining 211 Shasthya Sebikas were unable to attend and receive MNP. Many Shasthya Sebikas were unable to attend the training because they lived far away, their travel expenses exceeded the office’s travel allowance, and they had family issues.”* In-depth interview at the sub-national level, **IDI-07**

To overcome these challenges, BRAC issued a circular in September 2016, instructing each Shasthya Kormis to keep 40 boxes of MNP (equivalent to 1,200 sachets) for carrying and distributing to Shasthya Sebikas (who did not have MNP sachets) during their regular field visits. BRAC also issued another circular to local offices with instructions for distributing the expected or targeted amounts of MNP sachets (at least 6 boxes per Shasthya Sebika) and increase performance-based incentives for both Shasthya Sebikas and Shasthya Kormis to meet sales targets.

*“MNP shortages were observed among Shasthya Sebikas who did not visit BRAC’s local office to receive MNP or when BRAC’s local office was unable to supply MNP to these Shasthya Sebikas on time. Since 2016, we have reserved 40 boxes of MNP for each Shasthya Kormis allowing Shasthya Sebikas to receive MNP from the SK during her field visits. As a result, no MNP stockouts were observed among Shasthya Sebikas later.”* In-depth interview at the sub-national level**, IDI -09**

Four participants reported that the HF program staff struggled to distribute the expected number of MNP sachets to Shasthya Sebikas because other government and non-governmental organizations (NGOs) programs in their catchment area provided MNP for free. Shasthya Sebikas reported difficulties in selling MNP sachets as mothers preferred to receive MNP sachets at no cost from government healthcare facilities, leading to reduced demand for BRAC-supplied MNP.

*“Mothers are receiving free MNP sachets from government hospitals in my area at Upazila Sadar. When we went to their homes to sell our MNP, mothers used to tell us that we might get free MNP from government health care facilities, and that you probably got free MNP from BRAC, but you’re trying to sell it to us - they refused to buy MNP from us at the time. As a result, the MNP sachets in my possession would not have run out, and I would have been unable to obtain new MNP sachets from the BRAC local office to sell at the household level.”* In-depth interview at the community level**, IDI-05**

Seven participants stated that some BRAC sub-district offices were unable to distribute the required amounts of MNP sachets to Shasthya Sebikas, which affected the Shasthya Sebikas’ ability to meet their monthly sales targets on the BRAC Nutrition Programme Platform. Shasthya Sebikas at the BRAC Nutrition Programme Platform reported to have met their monthly MNP sales targets before the stipulated time due to their lower workload, knowledge proficiency, and strong counseling skills. In response to the early achievement of sales targets, participants suggested that increasing the supply of MNP sachets to these high-performing Shasthya Sebikas could prevent stockouts and ensure a continuous supply to meet household demand.

#### Key other enablers taken to address MNP supply chain barriers or challenges

Five participants mentioned that GAIN played an important role in overcoming MNP supply chain barriers through continuous technical assistance and monitoring of the supply chain system. This included overseeing the supply flow of MNP sachets from Renata limited to BRAC’s central warehouse and further distribution from BRAC’s central warehouse to BRAC field offices, ensuring a consistent MNP supply. GAIN regularly followed up with BRAC to collect real-time stock data, while Renata limited received stock updates from BRAC on the 10th and 25th of each month to ensure timely restocking and MNP supply. GAIN also worked closely with Renata limited to implement a monthly MNP delivery schedule, ensuring demand and supply fluctuations were addressed promptly. Participants mentioned that these measures were agreed upon during high-level supply chain management meetings held in early 2016, resulting in effective initiatives that ensured adequate and timely MNP supply across various levels (national, sub-national, and community) as outlined in Table 1.

**TABLE 1:** Initiatives to improve MNP supply chain processes.

Collaborative initiatives	Actions taken
Monthly MNP delivery plan (from Renata limited to BRAC and BRAC to Shasthya Sebikas)	(1) Renata limited followed a monthly schedule to deliver MNP to BRAC (2) Renata limited delivered 60% of monthly MNP requisition from BRAC within the first 10 days of the respective month and rest 40% delivered by day 25 of the same month (3) BRAC ensured MNP supply in all programme areas by the second week of the month (before Shasthya Sebikas refreshers training)
Regular monitoring and stock update	(1) BRAC central store sent real-time stock in and out updates to the Head Office (2) BRAC maintained a compiled stock update report on an excel sheet (3) BRAC sent the stock update report to GAIN and Renata limited on the 10th and 25th of each month
Renata limited updates BRAC regularly on MNP production and supply	Renata updated BRAC as soon as possible if there was any situation that hampered MNP production and supply due to scarcity of raw materials and shipment rejection in quality control test to make the following possible solutions to continue smooth sales: (1) MNP supply was prioritized only in HF program areas other than BRAC’s other areas. (2) Maintained sufficient buffer stock at the central and local levels to reduce stockout.

Participants from BRAC and Renata limited reported that they enforced strict storage and packaging protocols to maintain the quality of the MNP sachets (e.g., product damage) in order to prevent disruption in MNP supply chain. MNP sachets were stored in tightly sealed, moisture-proof packaging made from food-grade, heat-sealed laminate materials. This protected the sachets from nutrient degradation, particularly the oxidation of iron, and contamination, ensuring the product remained fresh and potent. The sachets were also packed in secondary box to prevent damage and moisture exposure, reducing the risk of clumping and ensuring the product’s potency remained intact.

MNP storage conditions were tightly regulated, with environmental controls (specifically for humidity) in warehouses to prevent product deterioration. The storage duration varied depending on the stage of the supply chain. In BRAC’s central warehouses and distribution centers, MNP sachets were stored for a maximum of 6 months with regular stock rotation to ensure older stock was used first. At the community level, where Shasthya Sebikas sold MNP sachets directly to households, the sachets were stored for 1–3 months, ensuring timely distribution and reducing the likelihood of expired stock. These packaging and storage measures significantly improved MNP coverage and effectiveness by preventing product degradation and minimizing MNP supply delays. The continuous efforts by BRAC, GAIN, and Renata limited to address supply chain challenges ensured that MNP sachets reached beneficiaries in a timely manner and remained effective upon use.

## Discussion

This process evaluation identified important barriers to MNP supply chain, including delayed raw materials shipments and procurement processes, low-quality materials, limited storage capacity in BRAC’s central warehouse, MNP transportation problems, and coordination gaps for MNP requisition between national and sub-national levels. To address these barriers, key strategic measures include creation of the buffer stocks of MNP sachets and hiring separate storage facilities; dedicated vehicles to transport MNP from national to sub-national levels; and hiring of supply chain and quality assurance officer. These strategic course-corrections during implementation of HF with MNP programe collectively helped to create a robust, responsive, consistent, and reliable MNP supply chain. By addressing both the national and sub-national MNP supply chain barriers through improved coordination, transportation solutions, and proactive stock management, the program was able to ensure the timely availability of MNPs at all levels. In subsequent paragraphs, we will first discuss national-level barriers to MNP supply chain followed by sub-national-level barriers of MNP supply chain.

The significant challenges of relying solely on imported readymade MNP products for the HF programme, particularly in terms of quality control and supply chain disruptions, were highlighted in earlier research (6, 17–20). These challenges included shipment cancelations, product expiry, and delays that jeopardized timely MNP supply. In light of these challenges, our study findings emphasize the importance of prioritizing local MNP production as a solution to minimize these risks associated with imported products and ensuring a more consistent MNP supply for the HF programme. Local MNP production not only minimizes the risks associated with dependency on international suppliers, but also provides greater control over quality and timely MNP supply. Moreover, our study findings indicate that the HF with MNP program implementers can effectively reduce stockouts and improve MNP sales and coverage by addressing supply chain obstacles, including delayed raw material shipments, procurement processes, and low-quality materials. This discussion presents the interplay between supply chain barriers and enablers that emphasize on how thoughtful and coordinated course corrections such as improved MNP raw material procurement strategies, buffer stock management, and local production initiatives—can significantly enhance the effectiveness of MNP supply chain by addressing the root causes of supply chain disruptions and maximizing their impact.

Drawing parallels with experiences documented in Vietnam, Ethiopia, Malawi and Rwanda (8, 13), highlighting common barriers to consistent MNP supply through local production, including packaging issues, low-quality raw materials, market instability, and a lack of incentives for local manufacturers. These barriers often arise in local production contexts, where infrastructure and market dynamics vary significantly from region to region, impeding a consistent supply of quality MNPs. Our study emphasizes the complex socio-political and economic, and programmatic factors affecting the MNP supply chain in Bangladesh, which includes delays in raw material procurement, limited storage capacity, political unrest, and transportation issues. Tailored solutions and adaptive approaches such as manufacturing large quantities of MNP sachets when raw materials were available, establishing a structured monthly production schedule, hiring separate storage facilities, and arranging dedicated MNP transport (with donor support) were facilitated to better navigate systemic MNP supply chain challenges and reduce stockout risks.

We found in LMICs including Bangladesh and Kenya (17–21) that the absence of robust monitoring mechanisms negatively impacts the performance of the MNP supply chain, which is consistent with our study results. Our study findings further emphasize how the lack of a dedicated supply chain focal person at the sub-national level can disrupt the timely distribution and availability of MNP sachets at the sub-national level. To address this challenge, the recruitment of Officer Supply Chain at sub-national levels proved to be a strategic and effective solution. These personnels played a crucial role in improving supply chain efficiency by monitoring stock availability at the sub-national level and providing real-time updates on inventory levels; generating accurate reports on MNP stock levels, enabling better decision-making and planning; facilitating communication between sub-national and national levels, ensuring timely updates on stock requisitions; ensuring timely replenishment by overseeing the requisition process from sub-national offices to BRAC headquarters; and resolving local supply issues by coordinating with BRAC staff at various levels, thereby minimizing disruptions. This strategic response significantly reduced subnational-level barriers and improved the overall performance of the MNP supply chain. Moreover, the shift from a top-down supply chain approach to a bottom-up requisition model, which reflected actual field demand, further optimized the MNP supply chain efficiency (22). This bottom-up approach played a pivotal role in meeting distribution targets and improving the availability of MNP at sub-national levels, particularly in the third year of HF with MNP program implementation. We think that the bottom-up approach is typically more effective for optimizing the MNP supply chain, as it aligns closely with actual demand, enhance responsiveness, reduce wastage, and improve local accountability. These benefits collectively contribute to a more resilient and efficient MNP supply chain, which is particularly valuable where local needs and conditions are dynamic.

This process evaluation identified important economic factors influencing the MNP supply chain at the sub-national level, particularly the financial challenges faced by Shasthya Shebikas. The inability of Shasthya Sebikas to pay cash for MNP sachets and the insufficient transportation allowances, which discouraged them from traveling to BRAC local offices, were significant barriers to ensuring a smooth supply chain. These constraints hindered the timely distribution of MNP to beneficiaries and threatened the effectiveness of the HF program. To address these challenges effectively, BRAC implemented several strategies to streamline the MNP supply chain. One crucial strategy involved providing performance-based rewards or incentives to both Shasthya Sebikas and their supervisor namely Shasthya Kormis. This strategy was instrumental in motivating Shasthya Sebikas and Shasthya Kormis to sell more MNP sachets, thereby earning additional income to reinvest in purchasing MNP from BRAC local offices. For Shasthya Sebikas, who are unpaid volunteers and often face financial hardships, these incentives were crucial for their livelihood, allowing them to continue participating in the program (22). To further enhance supply chain efficiency, BRAC introduced feedback loops between program implementers and decision-makers. These frequent feedback mechanisms allowed for real-time course corrections during program implementation, ensuring timely responses to emerging challenges and the ongoing refinement of the MNP distribution model. Such adaptability enabled the program to scale and replicate its successful strategies in other regions.

Overall, our study findings provided a clear pathway for how future research can build upon our findings, particularly through longitudinal assessments to track context-specific MNP supply chain that may impact on coverage, utilization, and outcomes like childhood undernutrition and anemia reduction. In terms of policy, policymakers can use this research evidence in strengthening MNP supply chain and supporting the development of more robust home fortification with MNP programs. This could significantly influence policy reforms aimed at improving the implementation of home fortification programs for MNP distribution. This study findings are critical for policymakers and program implementers, as it directly impacts the effectiveness of MNP interventions and their ability to achieve desired public health outcomes. Lastly, the practical implications for MNP program implementers are crucial. By focusing on ways to streamline the MNP supply chain, our study findings offer concrete steps that could lead to more effective and sustainable MNP supply chain for Bangladesh and other LMICs.

### Strengths and limitation of the study

This study utilized multiple research methods to collect data from a wide range of participants in order to ensure in-depth understanding of the MNP supply chain, covering different perspectives and experiences. By collecting data from diverse geographical and operational contexts, the study gathered insights relevant to specific sub-national challenges and successes. This specificity enhances the study’s applicability for tailoring interventions to particular regions. Despite this strength, one limitation was the inability to initiate the process evaluation on MNP supply chain from the beginning of the Home Fortification with MNP programme due to budget constraints. While the study’s context-specific insights are a strength, they may also limit the generalizability of findings to other regions or similar programs with different structures or supply chain dynamics.

## Conclusion

This process evaluation identified the barriers and enablers within the MNP supply chain and provides practical recommendations for enhancing the design and implementation of large-scale Home Fortification with MNP programs in Bangladesh. By identifying barriers and enablers in the MNP supply chain at both national and sub-national levels, the study emphasizes the need for adaptive strategies, effective coordination, and alignment of supply chain management with field realities to improve MNP distribution, sales, and coverage. Recruiting dedicated supply chain personnel, improving storage facilities, implementing performance-based incentives, and strengthening monitoring mechanisms, contributed to a more reliable supply of MNP. The study highlights the importance of real-time course corrections during program implementation to ensure uninterrupted MNP supply and improve household-level access. The findings are invaluable for stakeholders, offering essential guidance for scaling-up MNP interventions and building resilient supply chains in similar contexts, ultimately contributing to better public health outcomes.

## Data Availability

The raw data supporting the conclusions of this article will be made available by the authors, without undue reservation.
